# Selective shape control of cerium oxide nanocrystals for photocatalytic and chemical sensing effect†

**DOI:** 10.1039/c9ra01519a

**Published:** 2019-05-07

**Authors:** Nam-Woon Kim, Dong-Kyu Lee, Hyunung Yu

**Affiliations:** Korea Research Institute of Standards and Science (KRISS) Daejeon 34113 Republic of Korea peacewithu@kriss.re.kr; Korea Advanced Institute of Science and Technology (KAIST) Daejeon 34141 Republic of Korea; Chungbuk National University (CBNU) Cheongju 28644 Republic of Korea dklee@chungbuk.ac.kr

## Abstract

In this study, we report the precise shape control of crystalline cerium oxide, whose morphology changes between nanorods and nanoparticles in a short time. The proposed synthetic route of cerium oxide nanorods was highly dependent on the reaction time, and 10 min was determined to be the optimum synthetic condition. The cerium oxide nanorods were further converted into nanoparticles by the spontaneous assembly of cerium oxide nanoparticles into nanorods. The transmission electron microscopy results showed that the synthesized nanorods grew with high crystallinity along the 〈110〉 direction. The cerium oxide nanorods have been proven to be very efficient electron mediators for use as excellent photocatalytic materials and highly sensitive chemical sensors. The chemical sensor fabricated on a carbon paper substrate showed the high sensitivity of 1.81 μA mM^−1^ cm^−2^ and the detection limit of 6.4 μM with the correlation coefficient of 0.950.

## Abstract 2

Selective shape control of cerium oxide nanocrystals is suggested for superior photocatalytic and chemical sensing.

## Graphical abstract

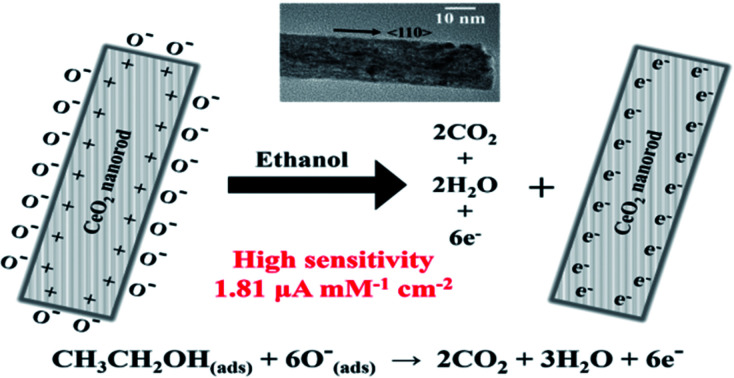

## 1. Introduction

The rapid increase in organic pollutants as a result of agriculture and large-scale industrial development has caused serious problems in the global scientific community. Due to their toxicities and persistence, organic pollutants esthetically contaminate, disturb, and eutrophicate aquatic organisms.^1–4^ Ethanol, a colorless liquid among a variety of organic pollutants, is prone to be released into the environment when abundantly used in the industry.^1,4,5^ Dyes have also been found in freshwater, industrial wastewater, and marine water because they have high and stable solubility.^6^ Ethanol can cause oxidative damage to the brain, stomach, liver, and red blood cells when it enters the body, whereas dyes exhibit the adverse effects of toxicity and carcinogenicity and are hazardous to the environment and human health;^1,4^ the effective detection of toxic ethanol and dyes in the environment is significantly important, and thus, a variety of methods have been reported in this regard.^7–12^ Previously, various chromatographic and spectroscopic techniques have been used for the detection of toxic solvents. However, these conventional techniques are often complex, slow, and difficult to be implemented in a wide range of applications. Therefore, electrochemical sensors that are simple, fast-responding, and capable of detection are the alternative candidates attracting significant attention.^13^ For the fabrication of efficient electrochemical sensors, it is important to recalibrate the material, size, structure, and properties of the electrodes. For application in the practical industry, it is essential to manufacture simple and low-cost sensors and active photocatalysts.^14,15^

Metal oxides such as ZnO and TiO_2_ are widely used in chemical sensors and organic contamination photocatalysts because their redox properties are related to the electron–hole pair on the metal oxide surface with analyte species.^3,4,14–25^ To improve the sensing and photocatalytic properties of nanomaterials, it is necessary to reduce the particle size and increase the active surface area of nanomaterials. Previous studies have shown that decreasing the particle size improves conductivity, electrical, sensing, and catalytic properties.^26–28^ In addition, one-dimensional (1D) nanomaterials, such as nanowires, nanorods, and nanotubes, exhibit significantly different physical and chemical properties from the bulk state due to their volume ratio and increased area for quantum confinement. They also form the building blocks for nanosized devices.^29–33^

Cerium oxide is a good material candidate for its well-known function in applications involving catalysts, sensors, optics, adsorption, electrochemistry, batteries, and energy storage.^6,34,35^ A variety of techniques have been used to synthesize nanoscale cerium oxide using surfactants or templates, such as precipitation, sonochemical, hydrothermal, reverse micelles, microemulsion, and sol–gel processes.^33,35–41^ Among these, hydrothermal synthesis is advantageous for applications in practical industries because simple processes can be easily scaled at a lower cost. Cerium oxide nanorods are efficient during CO oxidation, and they are often synthesized by the hydrothermal method with a high-pressure reactor (autoclave) at 100–200 °C for 10–72 h.^42–46^ These synthesis conditions are often difficult to emulate in the industry due to cost and time factors. Therefore, it is necessary to develop a very simple process that can be tactfully used in industrial applications at lower costs.

In this study, we investigated the morphology transformation of cerium oxide nanocrystals with the reaction time. Cerium oxide grows into nanorods and then rapidly reassembles into nanoparticles. Our cerium oxide nanorods exhibited worthwhile photocatalytic behavior over organic dyes and yielded a much-enhanced sensing effect as an ethanol sensor.

## 2. Experimental

### 2.1. Synthesis of cerium oxide nanocrystals

All the reagents are of the analytical grade and were used without further purification. The synthesis of cerium oxide nanorods is described in more detail in our earlier report.^47^ Cerium chloride (CeCl_3_·7H_2_O, 99.9%, Sigma Aldrich Co., Ltd.), ammonium chloride (NH_4_Cl, ≥99.5%, Sigma Aldrich Co., Ltd.), ammonia solution (NH_4_OH, 28.0–30.0%, Junsei Chemical Co., Ltd.), and deionized water were used to prepare the cerium oxide nanocrystals. Here, a 0.1 mol aliquot of CeCl_3_·7H_2_O and 1 mol of NH_4_Cl were added to a round-bottomed flask containing 20 mL of deionized water that boils at 100 °C. Then, 12 mL of boiling deionized water was added to 8 mL of ammonia solution under magnetic stirring. The diluent ammonia solution was directly injected into the cerium resource solution. It was confirmed that Ce(OH)_3_ sol was formed as soon as the solution was injected at the initial time of the reaction. Then, Ce(OH)_3_ sol rapidly converted into CeO_2−*x*_ (0 < *x* < 0.5) *via* Ce(OH)_4_ formation at the basic condition as Ce^3+^ is known to be less stable than Ce^4+^. The synthesis of cerium oxide nanoparticles varied depending on the reaction time of 1–20 min. The resulting precipitates were collected by centrifugation (2000 rpm, 5 min), washed with 100 °C deionized water, and dried at 60 °C for 24 h. The dried cerium oxide nanoparticles were calcined at 300 °C for 3 h (5 °C min^−1^). The color of the synthesized product was observed to be straw-yellow.

### 2.2. Characterizations of cerium oxide nanocrystals

The morphology of the cerium oxide nanocrystals was observed with an environmental scanning electron microscope (ESEM, Quanta 650 FEG, FEI Company) at an accelerating voltage of 30 kV. The samples for ESEM imaging were prepared by dispersing the cerium oxide nanocrystals in distilled water and drying them on a silicon wafer. Their chemical composition and weight were measured by energy-dispersive spectroscopy (EDS, ULTRA PLUS, Carl Zeiss NTS GmbH). A high-resolution transmission electron microscope (HRTEM, FEI Tecnai G2 F30 300 kV, FEI Company) was used to measure the intrinsic microstructure and intrinsic crystallographic properties. The crystallinity of the nanocrystals was determined using an X-ray diffractometer (XRD, SmartLab, Rigaku Co.). The sample was scanned with Cu-Kα radiation (*λ* = 1.5406 Å) at a rate of 0.2° s^−1^ to confirm the crystal structure, and the measured data were characterized by the Joint Committee on Power Diffraction Standards (JCPDS) database. The surface functional groups were observed with a confocal Raman microscope (Raman, DXR, Thermo Fisher Scientific). The granulation product was excited with a 532 nm laser and the spectrum was measured with a 50× objective combined with a pinhole (diameter: 50 μm). The X-ray photoelectron spectra (XPS, K-alpha, Thermo VG Scientific) were used to analyze the valence states of the cerium oxide nanocrystals. Ultraviolet visible spectroscopy (UV/Vis spectrophotometer, S-3100, Scinco Co.) was used to determine the optical properties of cerium oxide nanocrystals with large absorption in the ultraviolet (UV) region. The analytical method involved the total reflection of the powder samples using the integrating sphere (Integrating Sphere, Diffuse Reflector, Scinco Co.). The bandgap of the cerium oxides was determined from the measured data by the Kubelka–Munk function.

### 2.3. Photocatalytic experiment involving cerium oxide nanorods and nanoparticles

The photocatalytic decomposition of methyl orange was investigated by optical absorption spectroscopy. The photocatalytic reaction was carried out by mixing 20 mL of methyl orange solution (1.5 × 10^−4^ M, C_14_H_14_N_3_NaO_3_S, extra pure, Samchun Co., Ltd.) and 10 mg of cerium oxide nanorods in a 50 mL quartz beaker. The well-dispersed mixed solutions were irradiated using a 365 nm UV lamp (filtered UV lamp, VL-4.LC 4 W, Vilber Lourmat) and 2 mL samples were collected at 20 min intervals during irradiation. The collected methyl orange solution was separated from the cerium oxide nanorods *via* centrifugation. The UV/Vis absorbance was measured for the solution only after the nanorods were fully removed. The cerium oxide nanoparticles prepared after the synthesis time of 20 min were also analyzed under the same conditions for comparison.

### 2.4. Fabrication and characterization of ethanol chemical sensor

Ethanol chemical sensors were fabricated by coating the cerium oxide nanorods onto carbon electrodes (carbon paper, Alfa Aesar Co., Ltd.; surface area: 1 cm^2^). The carbon electrodes were cleaned with acetone, methanol, and isopropyl alcohol before coating. The cerium oxide nanorod powder was dispersed in a mixture of isopropyl alcohol, distilled water, and Nafion (Nafion D-521, Alfa Aesar Co., Ltd.) for coating. The prepared mixed solution was coated onto the carbon electrode of 100 °C by the drop-casting method before being dried at 70 °C for 6 h. To measure the sensing properties, the electrochemical cell was constructed using a simple two-electrode system (SP-150, Bio-Logic). A carbon electrode coated with cerium oxide nanorods was used as the working electrode and a Pt wire was used as the counter electrode. The current response was measured from 0.0 to +1.2 V. The prepared sensor was evaluated by adding various concentrations (0.17 to 85 mM) of ethanol (CH_3_CH_2_OH, ≥99.8%, Sigma Aldrich Co., Ltd.) to 20 mL phosphate buffer. The phosphate buffer solution was prepared by mixing 0.2 M sodium hydrogen phosphate (Na_2_HPO_4_, ≥99.0%, Sigma Aldrich Co., Ltd.) and 0.2 M sodium dihydrogen phosphate (NaH_2_PO_4_, ≥99.0%, Sigma Aldrich Co., Ltd.) solution in 200 mL deionized water. The prepared cerium oxide nanoparticles were also fabricated as a sensor and analyzed under the same conditions. Selectivity analyses (9100 HPLC system, Young Lin) and sensor characteristics for other pollutants (acetone, CH_3_COCH_3_, ≥99.9%, Sigma Aldrich Co., Ltd.) were performed for comparison.

## 3. Results and discussion

### 3.1. Growth process and properties of synthetic cerium oxide nanocrystals

**Fig. 1:**
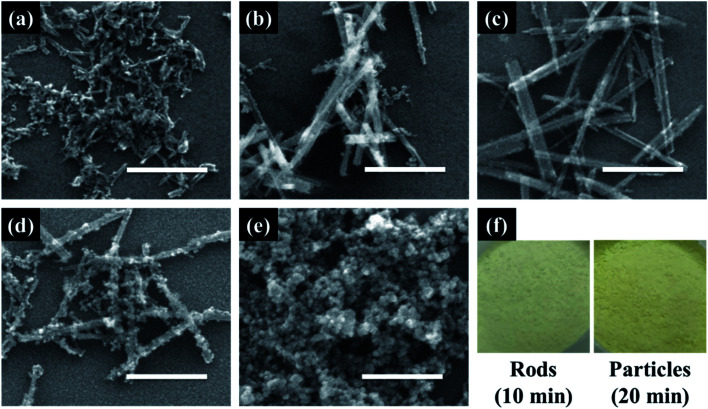
SEM images of cerium oxide synthesized at different reaction times of (a) 1 min, (b) 5 min, (c) 10 min, (d) 15 min, and (e) 20 min; (f) photographic image of cerium oxide nanorods and nanoparticles. Scale bar: 300 nm.

Cerium oxide nanocrystals were prepared by hydrothermal synthesis using an alkali solution. Pure cerium oxide powder is white; however, the color of the synthesized powder was straw-yellow and had a composition ratio of CeO_2−*x*_ (0 < *x* < 0.5). Fig. 1 shows a panorama of the ESEM images of the cerium oxide morphology with reaction time. After the initial nucleation, cerium oxide formed nanorods by self-assembly before becoming spherical particles. At a reaction time of 1 min, both spherical nanoparticles (size: ∼13 nm) and nanorods (length: ∼100 nm) were observed, as shown in Fig. 1a. After 5 min, long nanorods were prominent with some small debris of nanoparticles, as shown in Fig. 1b. After 10 min, definite cerium oxide nanorods had lengths between 400 and 800 nm with diameters of 20–50 nm, and the small spherical particles almost completely disappeared. It can be presumed that the small particles with the large surface energy per unit mass are more soluble than the larger particles, according to the Ostwald ripening phenomenon. Hence, large crystals continuously grow by consuming smaller crystals.^48–50^ As a result, our cerium oxide nanorods were fabricated after growth by the Ostwald ripening process. As shown in Fig. 1d and e, as the reaction time further increased to 15 min, it is apparent that smaller nanoparticles (15–25 nm) were attached to the rod surface. Finally, it was confirmed that cerium oxide was present only in the form of nanoparticles. It is assumed that metastable nanorods collapse to convert nanoparticles with minimal surface free energy.^51^ Fig. S1† shows the EDS results obtained to analyze the stoichiometric composition of the nanorods at a reaction time of 10 min. The precursor chemical species of Cl and NH_3_ were completely removed by washing, and pure Ce and O were monitored. From the EDS analysis, the composition ratio of the cerium oxide nanorods is expressed as CeO_1.8_ (Ce: 32.38 atom%; O: 58.17 atom%). This is evident as the straw-yellowish cerium powder in the optical images, as shown in Fig. 1f.

**Fig. 2:**
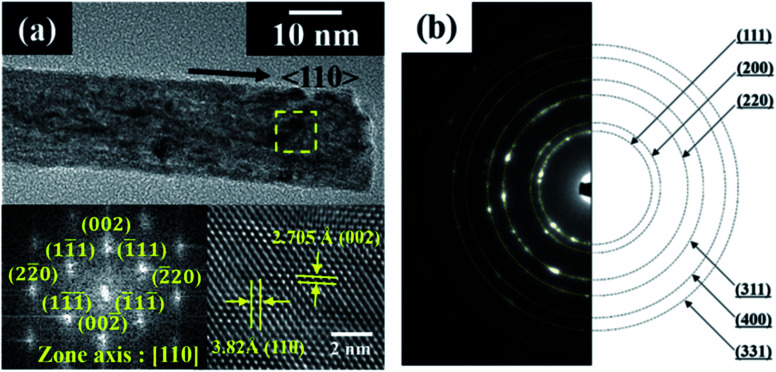
(a) HRTEM and FFT images and (b) SAED pattern of cerium oxide nanorods synthesized at 100 °C for 10 min.

Fig. 2 shows the HRTEM and fast Fourier transform (FFT) patterns of the synthesized cerium oxide nanorods. In the HRTEM image of Fig. 2a, the clear (110) and (001) lattice fringe directions, with interplanar spacings of 3.82 Å and 5.41 Å, respectively, show that the nanorods grew along the preferred growth direction, namely, 〈110〉.^34^ According to the FFT analysis, three kinds of lattice fringe directions, namely, (111), (002), and (220), of a typical nanorod were obtained. Fig. 2b shows the selected area electron diffraction (SAED) pattern recorded to investigate the crystal structure of the cerium oxide nanorods. The SAED pattern reveals several Debye–Scherrer diffraction rings that can be indexed to the crystal planes of a cubic fluorite structure with cerium oxide nanorods.

**Fig. 3:**
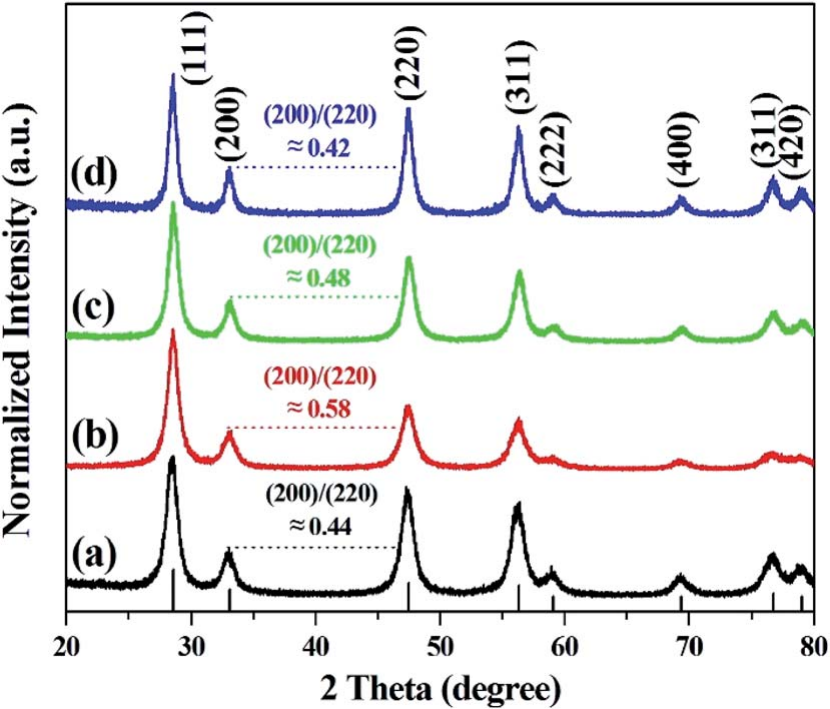
XRD patterns of cerium oxide at different reaction times of (a) 1 min, (b) 10 min (nanorod), (c) 15 min, and (d) 20 min (nanoparticle).

Fig. 3 shows the diffraction peaks in the XRD pattern of calcined cerium oxide, matching the reported values of the cerium oxide cubic fluorite structure with a lattice constant of *a* = 5.411 Å and space group *Fm3m* (JCPDS no.: 43-1002). The peak with the highest intensity is the (111) plane direction at 2*θ* = 28.5°. The crystallite size of cerium oxide was determined by the Debye–Scherrer equation with the highest intensity (111) peak along the reaction time, as summarized in Table S1.† It is interesting that the crystallite size increased from 79.7 to 113.7 nm with the reaction time, regardless of its morphological change, indicating particle growth and ripening. Here, we need to identify the growth direction during the shaping of the cerium oxide nanomaterial. In this characteristic peak, there are three kinds of planes: a stable and neutral plane (111), a stable plane (110), and a plane of high energy (001).^47^ The intensity ratio between (200) and (220) in the cerium oxide nanocrystal structure suggests the growth direction of the lattice parameter. The intensity ratio between (200) and (220) of the synthesized cerium oxide has a value between 0.42 and 0.58. The cerium oxide nanorods, with a reaction time of 10 min, show a ratio of approximately 0.58, which approaches the ideal value of 0.6.^47^ The nanorod crystallites tended to effectively grow along the (220) axis, which has also been observed for some other 1D materials.^47,50^ Cerium oxide was synthesized without other phases by matching the XRD pattern confirmed with the SAED pattern.

**Fig. 4:**
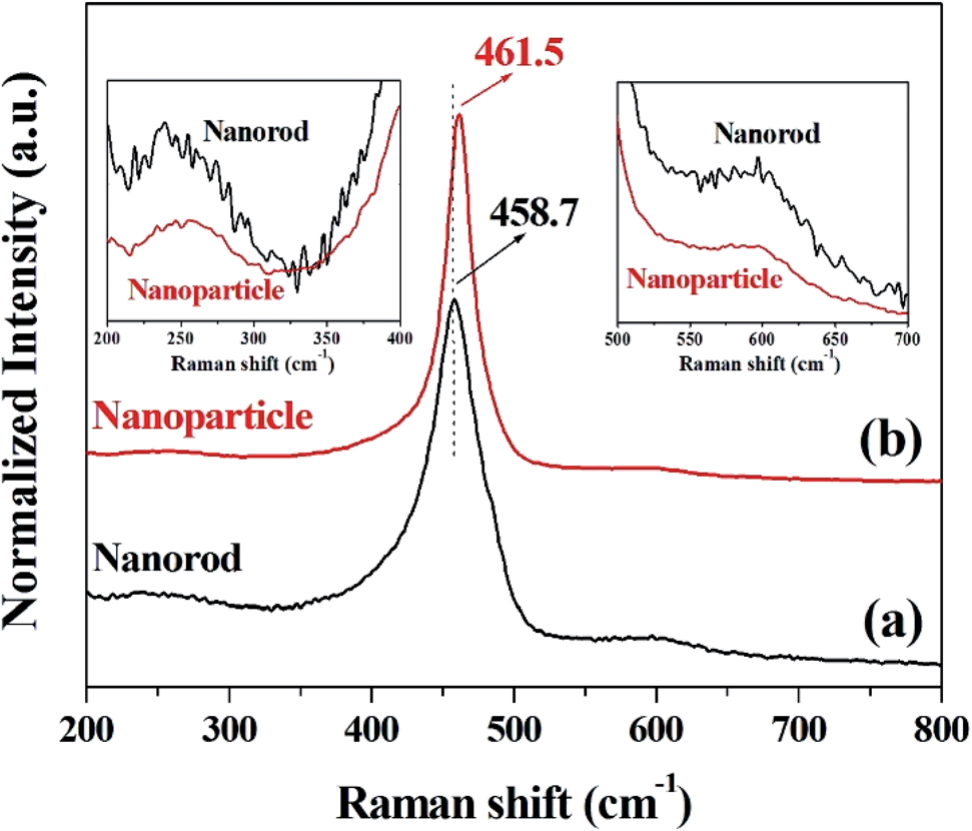
Raman spectra of cerium oxide at different reaction times of (a) 10 min (nanorod) and (b) 20 min (nanoparticle).

The crystallinity and structural information of cerium oxide were studied using a confocal Raman microscope. As shown in Fig. 4a and b, the symmetric stretching mode of the Ce–O vibrational unit (fluorite structure, F_2g_), which is the main peak of cerium oxide, was observed at 458.7 and 461.5 cm^−1^ (F_2g_ mode). In addition, we can observe the second-order transverse acoustic (2TA) mode (239.4 and 258 cm^−1^) and defect-induced (*D*) or oxygen vacancies mode (596.6 and 596.5 cm^−1^). An earlier study^47^ reported that the F_2g_ peak of cerium oxide exhibits a relatively low Raman frequency, which can result from the stabilizing effect of the oxygen structure in cerium oxide. As shown in Fig. 4, the intensities of the oxygen displacement and vacancy bands are higher for the cerium oxide nanorods than those of the cerium oxide nanoparticles. These trends are further revealed by the broadening of the F_2g_ mode around the 460 cm^−1^ band.^52^ A higher abundance of oxygen vacancies in the rod when compared with that of the particles indicates a more reducible surface oxygen species, which is the main factor for the photocatalytic behavior or chemical sensing.^52^ Fig. S2† shows the valence states of Ce and O ions in the cerium oxide nanocrystals for comparison with the oxygen vacancy rate on the surface. Both Ce and O ions have similar peak positions, as observed in earlier reports. The Ce 3d_5/2_ (882.1 eV) and Ce 3d_3/2_ (900.9 eV) peaks are assigned to the Ce^4+^ ion and Ce 3d_5/2_ (884.4 eV) and Ce 3d_3/2_ (903.0 eV) peaks are assigned to the Ce^3+^ ion. Further, it can be observed that the mixed valence state of Ce^4+^ and Ce^3+^ ions were present as satellites, and the ratio of Ce^3+^ peak area/Ce^4+^ peak area is summarized in Table S2.†^53^ As revealed by the Raman analysis, the XPS results also show that the cerium oxide nanorods have more oxygen vacancies on the surface than the cerium oxide nanoparticles.

**Fig. 5:**
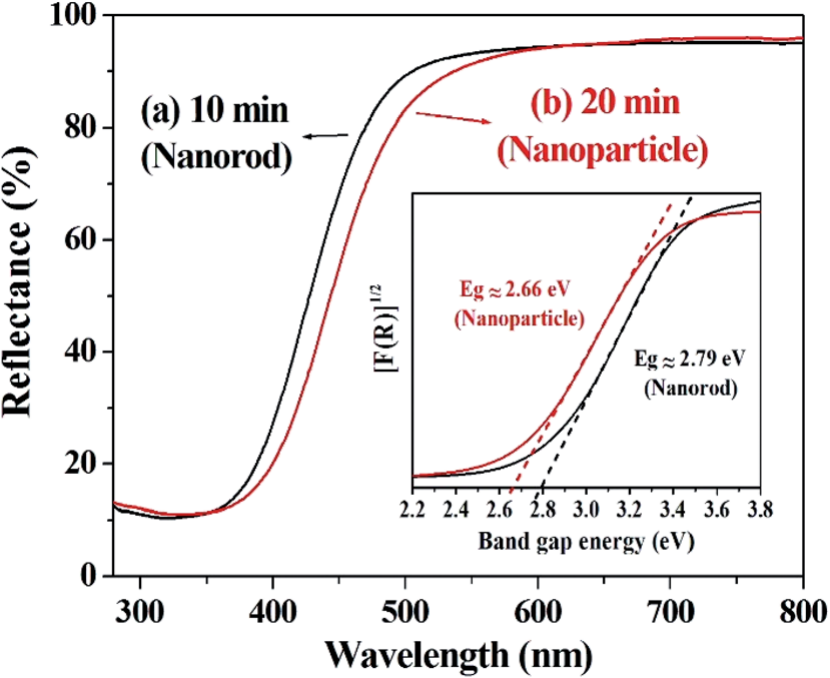
UV/Vis diffuse reflectance spectra of cerium oxide at different reaction times of (a) 10 min (nanorod) and (b) 20 min (nanoparticle). Inset: UV/Vis diffuse reflectance spectra plot as the Kubelka–Munk function.

The optical properties of the prepared cerium oxide were investigated using a UV/Vis reflectance spectrophotometer with an integrating sphere, as shown in Fig. 5. Absorption in the UV region of cerium oxide occurs by the charge transfer transition between the O 2p and Ce 4f states of O^2−^ and Ce^4+^, respectively.^51^ The synthesized cerium oxide showed a high absorption rate of approximately 90% in the UV region, while the reflectance was more than 90% in the visible-light region, yielding the straw-yellow color (Fig. 1f). As the reaction proceeded from 10 to 20 min, a notable red-shift (deep yellow) was observed due to the quantum confinement effect of excitons as the size of cerium oxide nanoparticle (crystallite size: 113.7 nm) became larger than that of the cerium oxide nanorod (crystallite size: 82.3 nm), as listed in Table S1.† In addition, cerium oxide inherently has a strong electron–phonon coupling effect; further, when the nanoparticle was more likely to aggregate than the rod, this coupling modified the effective mass of the carriers and scattering by the lattice, leading to a red-shift.^54^ The inset in Fig. 5 shows the calculated Kubelka–Munk function to measure the bandgap of cerium oxides. The bandgap was determined to be ∼2.79 eV for the nanorods and 2.66 eV for the nanoparticles, which is good for harvesting a wider range of light.

### 3.2. Photocatalytic degradation of methyl orange dye using cerium oxide nanorods and nanoparticles

**Fig. 6:**
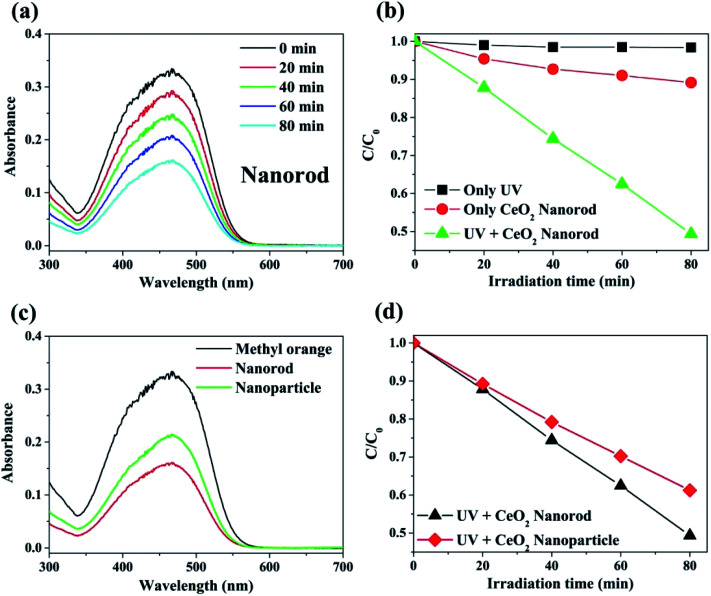
(a) Typical plot for the change in the absorption spectra on irradiation of an aqueous suspension of cerium oxide nanorod containing methyl orange (1.5 × 10^−4^ M) at various time intervals. (b) Plot for the degree of decomposition (*C*/*C*_o_) of methyl orange (1.5 × 10^−4^ M) with respect to UV exposure time. (c) Comparison of photocatalytic properties of cerium oxide nanorods with the nanoparticles (UV irradiation time: 80 min). (d) Decomposition degree of the methyl orange comparison with cerium oxide nanorod and nanoparticle with respect to UV irradiation time.

The photocatalytic activity of the prepared cerium oxide was investigated by performing decomposition of methyl orange under irradiation with 365 nm UV light, where the irradiation power was set to the minimum. The degradation rate of the methyl orange chemical species by cerium oxide nanorods was monitored by measuring the absorbance after every 20 min. Fig. 6a shows that the maximum absorbance of methyl orange dye at 461 nm gradually decreases with UV irradiation time. This phenomenon shows that the dye efficiently decomposed on the surface of the synthesized cerium oxide nanorods under UV irradiation. Furthermore, the cerium oxide nanorods after the photocatalytic reaction were fully separated by a centrifuge to identify no absorbance persisting in the range from 600 to 700 nm. Approximately 50% of the methyl orange decomposed after 80 min of UV irradiation. Fig. 6b shows a plot of the irradiation time *vs.* the relative concentration of the dye from the initial state during photolysis. No considerable decomposition can be observed when the methyl orange solution was exposed to UV irradiation without the cerium oxide nanorods. In addition, when only the cerium oxide nanorods were added to the methyl orange solution in the absence of UV irradiation, the absorption rate was 11.5% after 80 min. When the cerium oxide nanorods were exposed to light above the bandgap, electron and hole pairs were generated on the cerium oxide surface. Hydroxyl radical (OH˙) and superoxide radical anions (O_2_˙^−^) originated from the water in the oxygen in combination with the electron–hole pairs in the redox reaction, and they played a critical role in the oxidative reaction of the dye for photodecomposition.^6^Fig. 6c shows a comparison of the photocatalytic performance between the cerium oxide nanorods and cerium oxide nanoparticles when irradiated with 365 nm UV rays for 80 min. The nanorods show ∼11.9% higher photocatalytic activity than that of the nanoparticles, with less light absorbance in the UV region, as shown in Fig. 5. Meanwhile, the cerium oxide nanorods predominantly expose the reactive (110) and (100) planes, while the nanoparticles mainly expose the less reactive (111) plane on the surface. A typical cerium oxide nanoparticle comprises a polyhedron with eight (111) and six (100) planes, while the rod structure prefers to expose four (110) and two (100) planes. The oxygen is confined mainly on the surface of the nanoparticle, but it is occupied both on the surface and bulk so the nanorod provides a much higher oxygen capacity to significantly enhance the redox property. Therefore, the higher reactive plane and well-defined active sites induce additional catalytic behavior with less UV absorbance.^44,55^ In addition, the geometrical 1D structure of cerium oxide nanorods enables fast and long-distance electron transport.^56^ We conclude that cerium oxide nanorods decompose methyl orange more effectively than cerium oxide nanoparticles due to shape and structural advantages, as shown in Fig. 6. The stability of cerium oxide nanorods as a photocatalyst was also confirmed, as shown in Fig. S3.† A cyclic degradation experiment was performed using cerium oxide nanorods fully separated by a centrifuge after 80 min of UV irradiation. The moiety of methyl orange was adsorbed on the surface of the cerium oxide, which decomposed 4% less than the first decomposition rate. The cerium oxide nanorods exhibited good photocatalytic performance even after decomposition was repeated for three times.

### 3.3. Ethanol chemical sensor using cerium oxide nanorods and nanoparticles

**Fig. 7:**
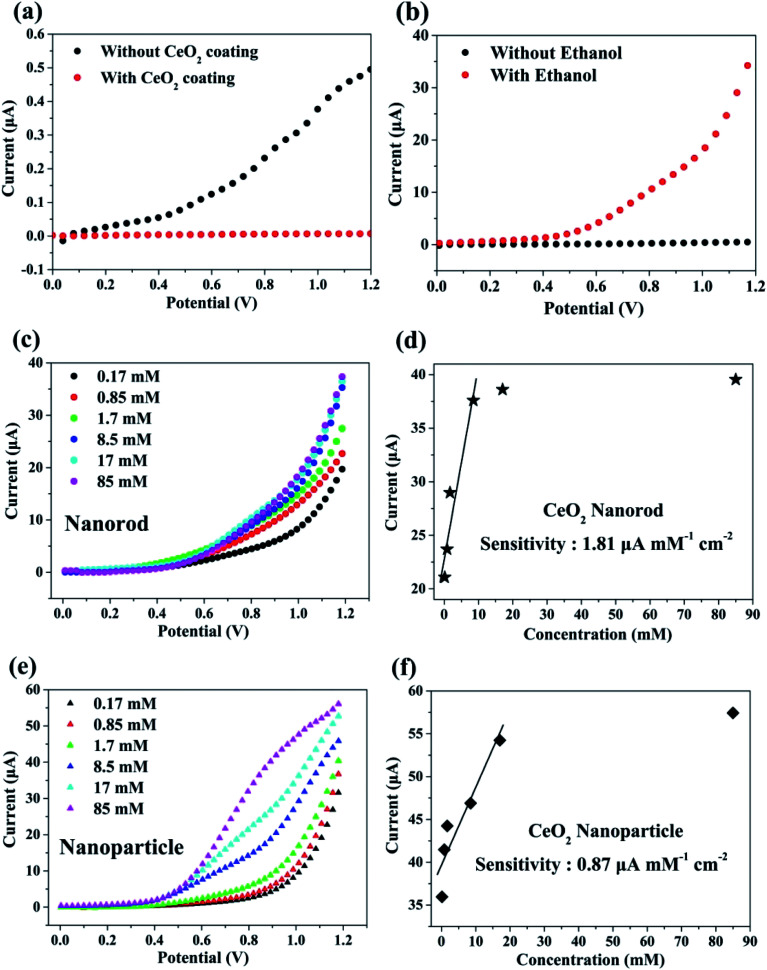
*I*–*V* characteristics of cerium oxide: (a) comparison of the current with and without cerium-oxide-nanorod-coated carbon paper. (b) Current test with ethanol (17 mM) in the cerium oxide nanorod. (c) Plot of the sensing behavior by a series of ethanol concentrations in the cerium oxide nanorod. (d) Calibration plot to deduce ethanol sensitivity of the nanorod. (e) Plot of the sensing behavior with different concentrations of ethanol in the cerium oxide nanoparticle. (f) Calibration plot to deduce ethanol sensitivity of the nanoparticle.

To monitor the sensing behavior, the synthesized cerium oxide was coated onto carbon paper, functioning as a working electrode. To analyze the sensing parameters of the electrochemical sensors, the current–voltage (*I*–*V*) curves were determined for a 0.1 M phosphate buffer solution (PBS, pH = 7.0) at various ethanol concentrations ranging from 0.17 to 85 mM. Fig. 7a shows the electrochemical effect of the carbon paper substrate on the *I*–*V* curve. The current decreased after the coating of cerium oxide nanorods (red circle) onto the substrate as compared to pure carbon paper (black circle). It is natural that the resistance increases by the nanomaterials mixed with the conductive binder uniformly coated on the sensor surface.^4^Fig. 7b shows the current change in the electrochemical response as a function of the working potential when exposed to ethanol for the fabricated chemical sensor. There appears to be no current response in the pure PBS solution, but the electrical response significantly increased with the addition of ethanol. This phenomenon can be explained by the rapid electron exchange and excellent electrocatalytic oxidation properties of the cerium oxide nanorods, which are suitable for ethanol-sensing applications. Generally, ethanol is subjected to a two-step process in the *I*–*V* analysis to determine its conductivity. The first step involves dehydrogenation, or dehydration, to produce CH_3_CHO (reaction (1)) and C_2_H_4_ (reaction (2)) as the respective intermediates.

1 C_2_H_5_OH → CH_3_CHO + H_2_

2 C_2_H_5_OH → C_2_H_4_ + H_2_O

In the second step, the resulting CH_3_CHO and C_2_H_4_ are oxidized by oxygen molecules physically adsorbed on the surface to yield CO_2_, CO, and H_2_O.^4,57^Fig. 7c shows the electrical response of cerium oxide nanorod thin films by adding 0.17–85 mM of various ethanol concentrations to a 0.1 M PBS solution. Each experiment was conducted by preparing a 0.1 M PBS solution containing a fresh cerium oxide nanorod electrode and fresh ethanol. The current of the working electrode increased with the concentration of ethanol. Thus, the conductivity of the cerium oxide nanorod electrode increased according to the concentration of the target chemical (EtOH). Fig. 7d shows a calibration curve plot determined from the data shown in Fig. 7c. The modified cerium oxide nanorod chemical sensor shows high sensitivity of 1.81 μA mM^−1^ cm^−2^, detection limit of 6.4 μM, and correlation coefficient (*R*) of 0.950. The calibration curve (Fig. 7d) shows a low concentration range up to 8.5 mM with good linear correlation of 95%. The prepared cerium oxide nanorod chemical sensor electrode exhibits an increase in current with increasing ethanol content up to 8.5 mM, followed by saturation. This can be attributed to the low availability of free active sites because the concentration of ethanol is higher than the active surface of the cerium oxide nanorod electrode for the adsorption of ethanol. Oxidation reactions using cerium oxide involve the participation of lattice oxygen species.^58^Fig. 7e shows the electrical response of cerium oxide nanoparticles measured for comparison with the cerium oxide nanorods used as chemical sensors. For comparison, the experiment was also conducted under the same conditions as the nanorods using 0.1 M PBS solution. By increasing the concentration of ethanol from 0.17 mM to 85 mM, the current at the working electrode of cerium oxide nanoparticles increased. Fig. 7f shows the calibration curve plotted using the data shown in Fig. 7e. The fabricated cerium oxide nanoparticle chemical sensor shows sensitivity of 0.87 μA mM^−1^ cm^−2^, detection limit of 14.7 μM, and *R* of 0.93. The calibration curve shown in Fig. 7f has a concentration range up to 17 mM and *R* of 0.93. Earlier reports^42^ have shown that cerium oxide nanorods have better CO oxidation performance with lower specific surface areas than the nanoparticles. This originates from the different reactivities along the crystal facets. The cerium oxide nanorods prefer to grow along the (110) and (100) planar surfaces of high activity, while cerium oxide nanoparticles grow along the (111) plane, which is less reactive.^42^ Many theoretical calculations have shown the oxidation reactivity follows the order: (100) > (110) > (111).^44,45^ Therefore, a chemical sensor performance accompanied by an oxidation reaction is highly dependent on the crystal facet exposed to the particle surface.^59,60^ It has also been reported that a reduced cerium oxide (CeO_2−*x*_) structure exhibits high photocatalytic efficiency due to reoxidation resulting from the unstable oxygen vacancies.^61^ In summary, our synthetic cerium oxide nanorods show high sensitivity as compared to cerium oxide nanoparticles. Moreover, the consequent chemical sensors fabricated using cerium oxide nanorods have higher sensitivity than those proposed in earlier reports (Table S3†)^62^ using cerium oxide nanoparticles and are successfully investigated as electrodes. Fig. S4† shows the sensing properties of cerium oxide nanorods for other pollutants such as acetone, which is an excellent chemical sensor for ethanol. For comparing the sensing performances, acetone was also added in the same volume ratio as ethanol under the same conditions as those used in the ethanol experiment. The cerium oxide nanorod sensor showed sensitivity of 1.99 μA mM^−1^ cm^−2^, detection limit of 11.68 μM, and *R* of 0.81. To determine the contamination selectivity of the cerium oxide nanorod chemical sensor, we operated the chemical sensor with the same volume of ethanol and acetone in a 0.1 M PBS solution. It was confirmed that the sensitivity of cerium oxide nanorods toward acetone is higher than that toward ethanol, as shown in Fig. S5.† The different sensing behaviors suggest that the fabricated cerium oxide nanorod chemical sensor can detect other pollutants in water with good selectivity.

**Fig. 8:**
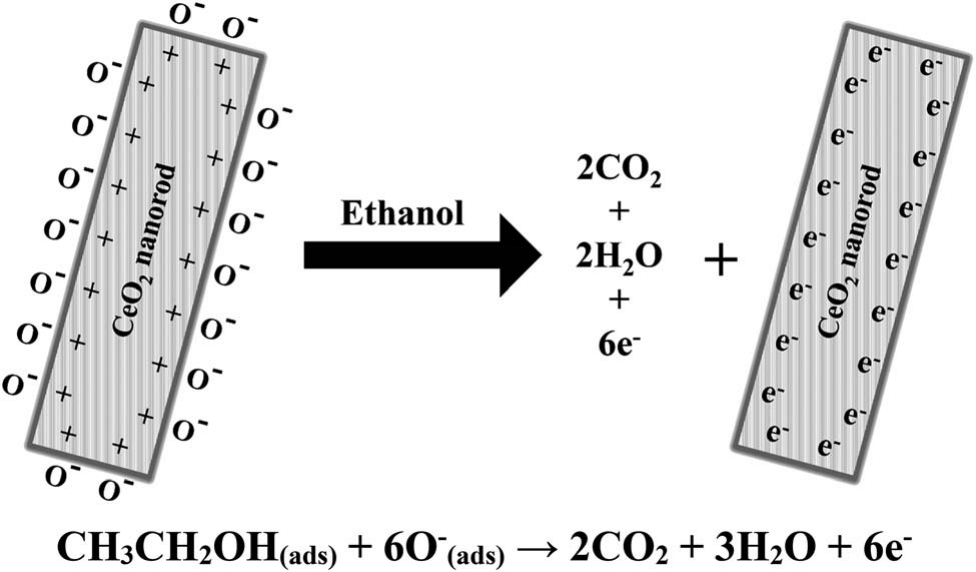
Proposed mechanism of ethanol sensing in the presence of cerium oxide nanorods.

A schematic mechanism of ethanol sensing with cerium oxide nanorods is shown in Fig. 8. As compared to the other forms of cerium oxide, the enhanced catalysis of rods is strongly influenced by the crystal plane and geometrical structure. The structural sensitivity of rods can be explained by the unique surface structure of the (110) and (100) planes, which increases the oxygen diffusion rates through the 1D cerium oxide axis.^44^

## 4. Conclusion

In summary, we successfully controlled the shape of cerium oxide nanocrystals by controlling only the reaction time without necessitating the use of a high-pressure reactor. The cerium oxide nanorods dramatically changed in shape into nanoparticles in only 10 min. The synthesized cerium oxides are present in a composition of CeO_2−*x*_ (0 < *x* < 0.5) and exhibit bandgaps of 2.79 eV and 2.66 eV for the nanorod and nanoparticle, respectively. The photocatalytic properties of cerium oxide were monitored by the decomposition of methyl orange. The results show that by using cerium oxide nanorods, 50% of the methyl orange dye was degraded in 80 min by simple exposure to UV light at 365 nm. The production of chemical sensors to detect ethanol was successfully accomplished using a substrate made of carbon paper. The fabricated cerium oxide nanorod chemical sensor exhibits high sensitivity of 1.81 μA mM^−1^ cm^−2^, detection limit of 6.4 μM, and *R* of 0.950. The cerium oxide nanorods were approximately twice as sensitive as cerium oxide nanoparticles. This indicates that the morphology control of cerium oxide is critical in chemical sensor applications. We have developed a novel synthesis method to fabricate cerium oxide nanorods that can be beneficial for mass production at lower costs in industrial applications. The synthesized cerium oxide nanorods are suitable for the application of photocatalysts for organic dye decomposition and are effective and sensitive chemical sensors.

## Conflicts of interest

There are no conflicts to declare.

## Supplementary Material

RA-009-C9RA01519A-s001
