# Supplementary material for: Selective shape control of cerium oxide nanocrystals for photocatalytic and chemical sensing effect
Source: RSC Adv. 2019 May 7;9(24):13829–37. doi: 10.1039/c9ra01519a (PMC9064037; doi:10.1039/c9ra01519a)
Supplement: RA-009-C9RA01519A-s001 [file RA-009-C9RA01519A-s001.pdf]

Electronic Supplementary Material (ESI) for RSC Advances.
This journal is © The Royal Society of Chemistry 2019

Supplementary Information

Selective Shape Control of Cerium Oxide Nanocrystals for Photocatalytic and Chemical Sensing Effect

Nam-Woon Kim,[ab] Dong-Kyu Lee,[*c] Hyunung Yu,[*a]

a. Industrial Metrology, Korea Research Institute of Standards and Science (KRISS), Daejeon 34113, Republic of Korea. Email : peacewithu@kriss.re.kr

b. Department of Materials Science and Engineering, Korea Advanced Institute of Science and Technology (KAIST), Daejeon 34141, Republic of Korea.

c. Department of Engineering Chemistry, Chungbuk National University, Cheongju 361-763, Republic of Korea. Email : dklee@chungbuk.ac.kr

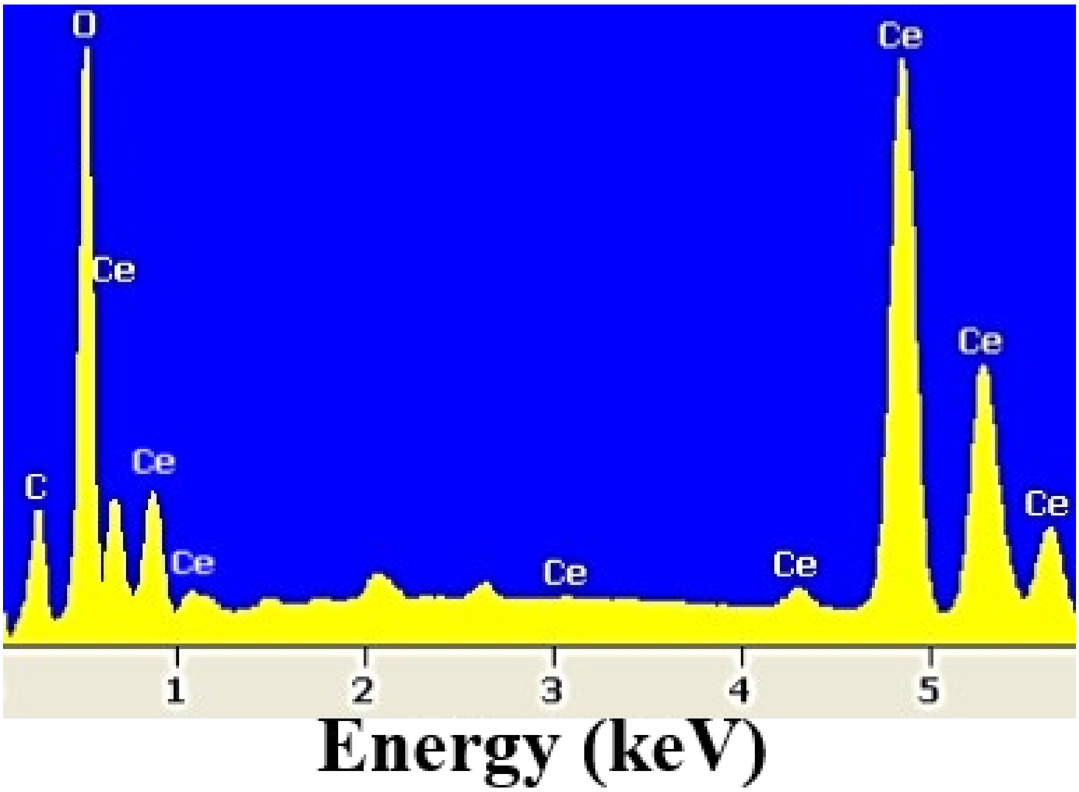

Fig. S1. EDS of cerium oxide synthesized at 10 min (nanorod).

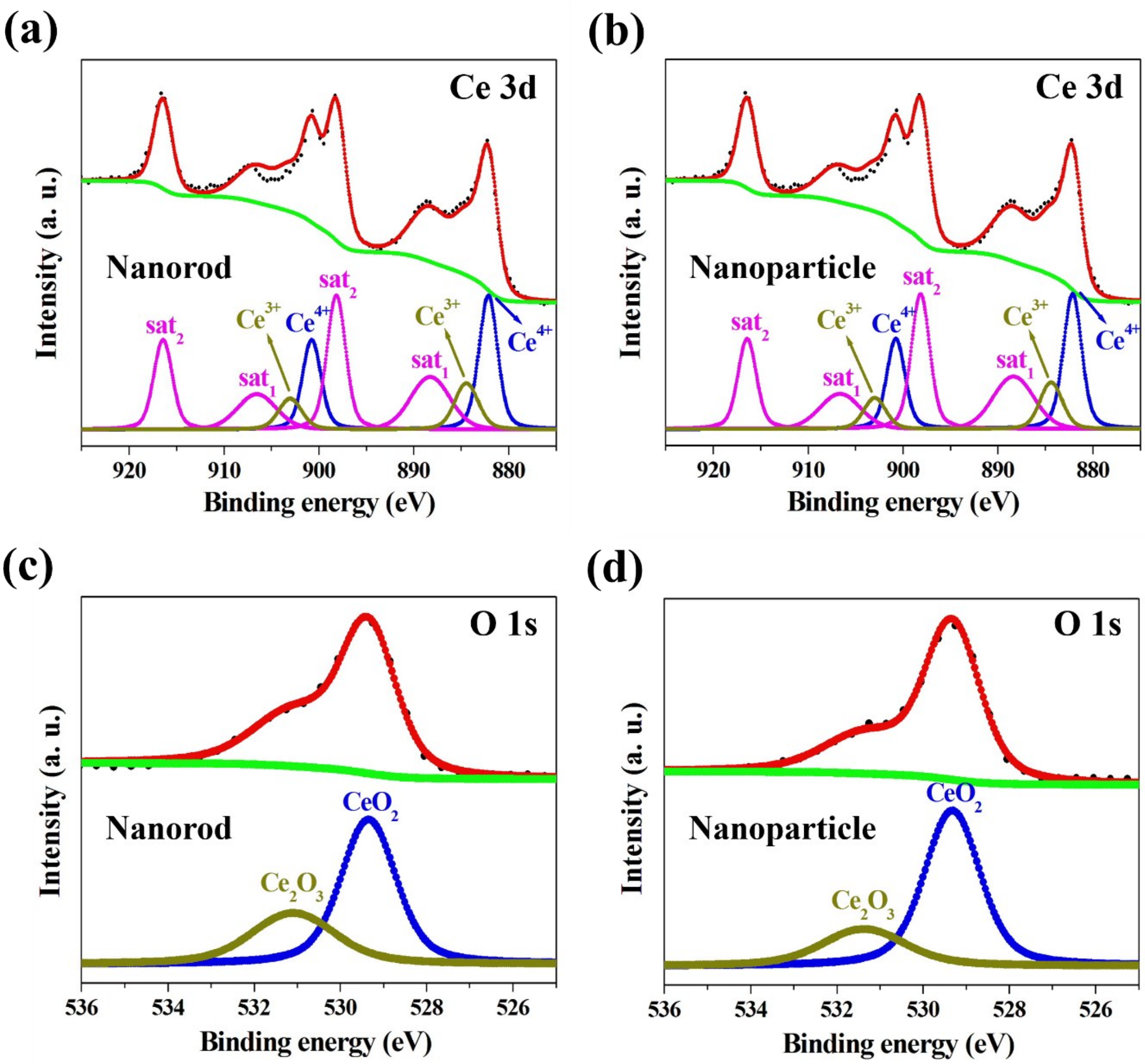

Fig. S2. XPS spectra of cerium oxide synthesized at 10 min (nanorod: a, c) and 20 min (nanoparticles: b, d).

Upper; Black dot - Raw data, Red line – Fitting curve with green base line

Lower; Deconvoluted peaks with satellite peaks (violet)

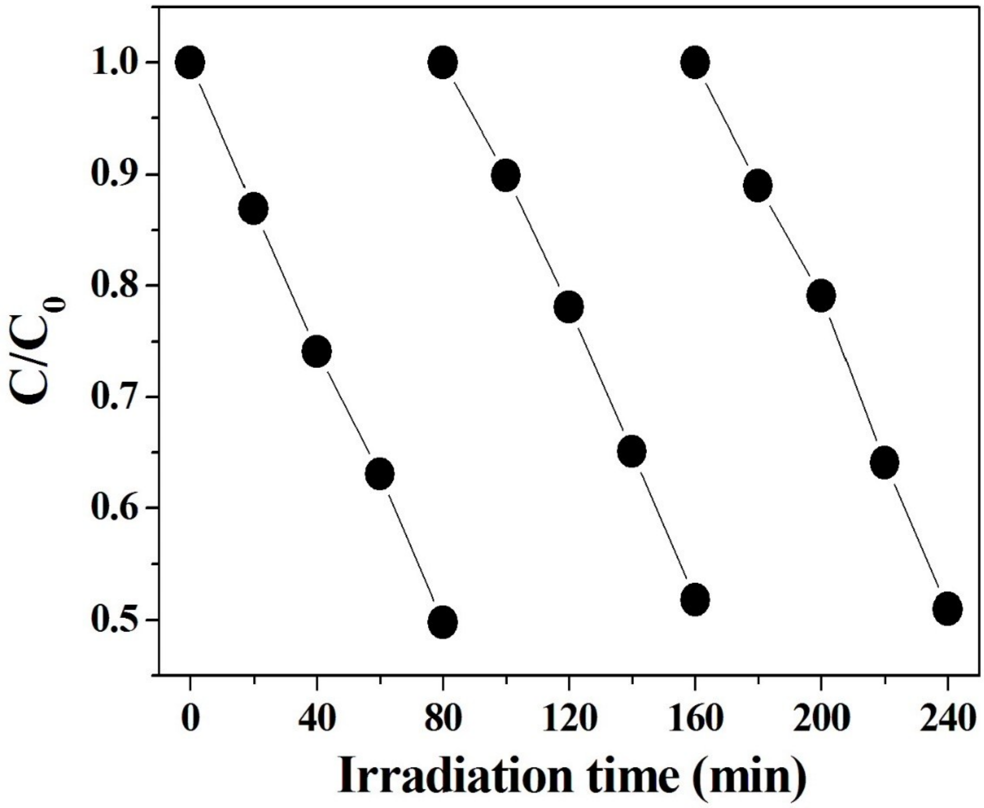

Fig. S3. Cycling performance of cerium oxide nanorod during the photodegradation of methyl orange.

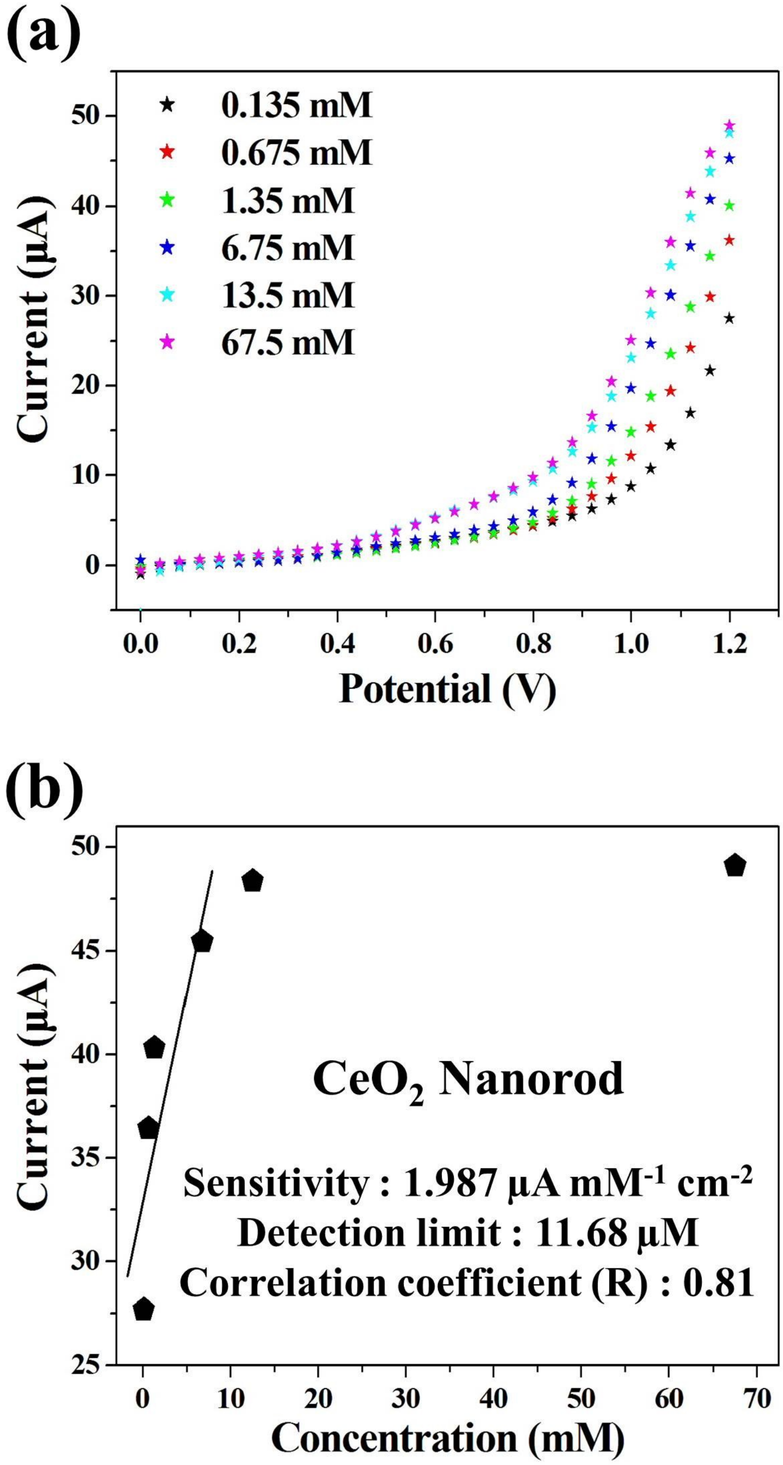

Fig. S4. I-V characteristics of cerium oxide nanorod: (a) Plot of the sensing behavior by a series of acetone concentration in the cerium oxide nanorod; and (b) Calibration plot to deduce an acetone sensitivity of nanorod.

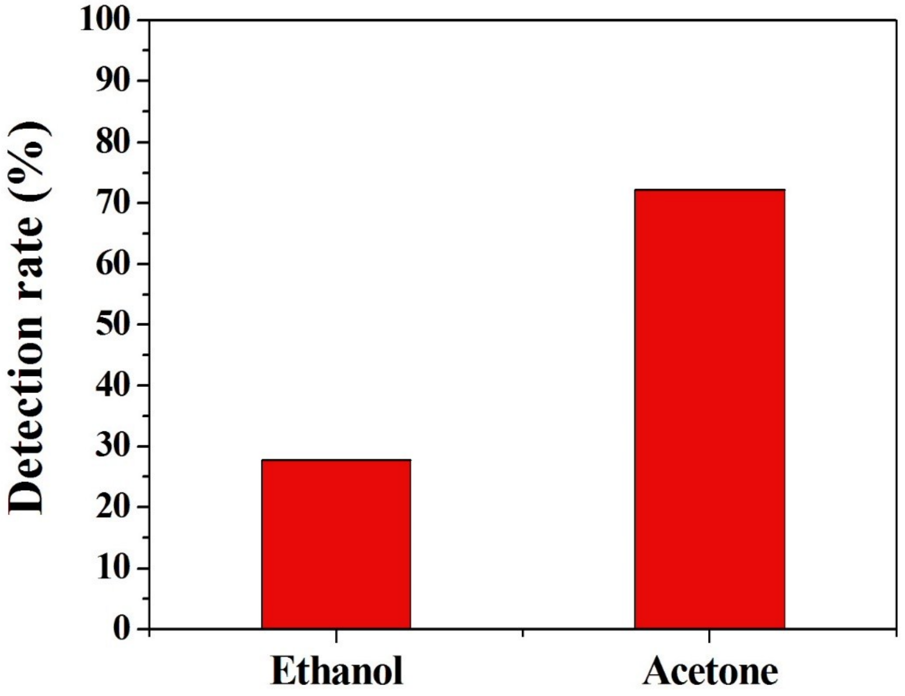

Fig. S5. Percentage of contaminants detected using cerium oxide nanorod sensors.

Table S1. The cerium oxide crystallite size calculated based on Scherrer equation and the intensity ratio of (200)/(220) with the reaction time using the XRD patterns. ([a] Full Width at a Half Maximum)

Reaction time	FWHM[a]	Crystallite sizes (nm)	(200)/(220)
1 min	1.074	79.7	0.44
10 min	1.042	82.3	0.58
15 min	0.937	91.3	0.48
20 min	0.753	113.7	0.42

Table S2. XPS analysis result of Ce^{3+} and Ce^{4+} ion concentration.

Reaction time	Ce^{3+} peak area/Ce^{4+} peak area
10 min (Nanorod)	0.43
20 min (Nanoparticle)	0.33

Table S3. Comparison of photo-catalytic and chemical sensor performance of various materials reported in previous literature.

Material	Shape	Dye decomposition for 80 minutes(%)	Chemical sensor sensing material	Sensitivity ($\mu A\ mM^{-1}\ cm^{-2}$)	Reference
CeO_2	Nanorod	50	Ethanol	1.81	Present work
CeO_2	Nanoparticle	38	Ethanol	0.87	Present work
CeO_2	Nanorod	-	Acetone	1.99	Present work
CeO_2	Nanoparticle	26	Ethanol	0.92	Ref 4
CeO_2	Nanoparticle	28	Ethanol	1.19	Ref 6
CeO_2	Nanoflake	78	hydroquinone	2.04	Ref 35
Cobalt-doped CeO_2	Nanorod	39	-	-	Ref 33
CuO	Nanosheet	-	Ethanol	0.9722	Ref 1
ZnO–CeO_2	Nanomaterial	70	Ethanol	2.1949	Ref 3
Polyaniline grafted graphene oxide-WO_3	Nanocomposite	-	Chromium (III)	4.4251	Ref 62
